# Development and initial validation of the Comprehensive Geriatric Oral Health Assessment Tool

**DOI:** 10.1002/cre2.791

**Published:** 2023-09-28

**Authors:** Shervan Shoaee, Mohammad‐Hossein Heydari, Hossein Hessari, Neda Mehrdad, Laleh Khalilazar, Bahareh Hatami, Farshad Sharifi

**Affiliations:** ^1^ Elderly Health Research Center, Endocrinology and Metabolism Population Sciences Institute Tehran University of Medical Sciences Tehran Iran; ^2^ Non‐Communicable Diseases Research Center, Endocrinology and Metabolism Research Institute Tehran University of Medical Sciences Tehran Iran; ^3^ Research Center for Caries Prevention, Dentistry Research Institute Tehran University of Medical Sciences Tehran Iran; ^4^ School of Dentistry Shahid Beheshti University of Medical Sciences Tehran Iran; ^5^ Endocrinology and Metabolism Research Center, Endocrinology and Metabolism Clinical Sciences Institute Tehran University of Medical Sciences Tehran Iran

**Keywords:** aging, geriatric, oral health assessment, tool

## Abstract

**Objectives:**

Improving the oral health of the elderly is crucial to improving their general health and quality of life. To reach this goal, it is necessary to start with a comprehensive oral health assessment and a detailed treatment plan. The aim of this study was, therefore, to develop a comprehensive Oral Health Assessment Tool for the geriatric population.

**Material and Methods:**

Following a panel of experts' consultation, a clinical form and a self‐assessment questionnaire were developed, encompassing eight domains: dental caries, periodontal diseases, partial and complete edentulism, oral soft tissue lesions, occlusion, xerostomia, temporomandibular joints, and oral or oral prostheses hygiene. Subsequently, a pilot study was conducted to appraise the clinical form and questionnaire involving 84 residents of an Iranian nursing home. After securing ethics approval, both the qualitative and quantitative aspects of the self‐assessment questions' validity and reliability were assessed, and specificity and sensitivity were calculated.

**Results:**

The mean age of the participants was 69.8 (±4.1) years, and 86% had less than 12 years of education. The questions regarding the number of remaining teeth and the number of decayed teeth had the highest sensitivity (97% and 88%), respectively. Questions regarding the presence of periodontitis and gingivitis had the highest specificity (both 100%).

**Conclusions:**

A Comprehensive Geriatric Oral Health Assessment Tool has been developed and its validity and reliability evaluated in a pilot study. It should now be further evaluated in larger studies.

## 1 INTRODUCTION

Inadequate oral health can exert detrimental effects on overall well‐being and is associated with systemic conditions, including diabetes, cardiovascular diseases, malignancies, obesity, and pulmonary conditions (Ahmad & Haque, 2021; De Angelis et al., 2018; Baldomero et al., 2019; Corbella et al., 2018; Deshpande & Amrutiya, 2017; Shoaee et al., 2023; Shoaee, Saeedi Moghaddam, et al. 2022; Sofi‐Mahmudi et al., 2021). An increasing number of more than 3.5 billion individuals are grappling with oral health‐related issues. Edentulism, periodontal diseases, and dental caries are among the most prevalent oral disorders on a global scale (Bernabe et al., 2020; Moscowchi et al., 2023; Peres et al., 2019; Watt et al., 2019). Beyond the substantial burden imposed by these conditions, their economic ramifications cannot be disregarded. An estimate suggests that the direct and indirect costs attributed to oral diseases reached 442 million in 2015 (Listl et al., 2015).

The issue of multimorbidity is becoming ever more important with the increase in life expectancy and the subsequent demographic transition (Immonen et al., 2020). Among different age groups, the geriatric population is at higher risk for chronic diseases and chronic oral health conditions such as periodontitis, edentulism, and dental/root caries (Barrio‐Cortes et al., 2021; Gil‐Montoya et al., 2015; Mortazavi et al., 2023). The prevalence and burden of untreated caries, severe periodontitis, and tooth loss have increased from 1990 to 2015, and more importantly, these conditions affect the elderly population more severely (Kassebaum et al., 2017). The initial efforts on improving the oral health of the elderly was begun in 2005 by the World Health Organization's (WHO) global oral health program (Petersen & Yamamoto, 2005). Assessment of oral health and oral health‐related quality of life is a crucial and necessary step in improving the oral health of this population. Several Oral Health Assessment Tools (OHATs) were developed to evaluate different aspects of oral health status and oral health‐related quality of life among the elderly (Adulyanon, 1997; Adulyanon et al., 1996; Anagnostopoulos, 2014; Atchison & Dolan, 1990; Chalmers et al., 2005; Kayser‐Jones et al., 1995; Leao & Sheiham, 1995; Petersen & Baez, 2013); however, a comprehensive tool that encompasses these aspects is still lacking. This study aims at developing a new comprehensive tool to tackle this problem.

## 2 MATERIALS AND METHODS

### 2.1 Concept analysis and framework of study

Initially, we conducted searches in the PubMed, EMBASE, Web of Science, and Scopus databases using relevant keywords to identify available Geriatric Oral Health Assessment Tools. This search obtained six questionnaires designed to assess the oral health status of the geriatric population (Adulyanon, 1997; Adulyanon et al., 1996; Anagnostopoulos, 2014; Atchison & Dolan, 1990; Chalmers et al., 2005; Kayser‐Jones et al., 1995; Leao & Sheiham, 1995; Petersen & Baez, 2013). Following this, an expert panel consisting of specialists in community oral health, oral medicine, and geriatric medicine was convened to assess and evaluate these questionnaires. The consensus from the panel was that there is a lack of a comprehensive questionnaire for evaluating the oral health status of the elderly. These identified questionnaires primarily focused either on assessing specific aspects such as occlusal, aesthetic, or verbal functions or on evaluating the oral health‐related quality of life among the geriatric population. Although the WHO Oral Health Survey was recognized as one of the most comprehensive, it still exhibited certain limitations in its domains when applied to the geriatric population. Furthermore, it primarily constituted a clinical evaluation of oral health and did not encompass the individual's self‐assessment of their oral health status or their level of oral health satisfaction.

Second, an expert panel was formed, which included 10 experienced dentists and physicians in the field of community oral health, prosthodontics, oral medicine, epidemiology, and geriatric medicine. After a thorough discussion of the crucial issues regarding the oral health status of the geriatric population, eight domains—based on the domains that were previously introduced by WHO oral health survey (Petersen & Baez, 2013)—were selected for the development of the new tool, including dental caries, periodontal diseases, edentulism and tooth loss, oral lesions, occlusal function, xerostomia, temporomandibular joint (TMJ), and oral or prosthetic hygiene.

### 2.2 Tool development and validity

Our intention was to develop two distinct forms for assessing the oral health of the geriatric population:

- A self‐assessment questionnaire designed to evaluate an individual's subjective oral health.
- A clinical evaluation form is to be completed following an oral examination by trained dentists, providing an overview of the individual's oral health status.

The self‐assessment form could also serve as a data collection tool in situations where a comprehensive oral and dental examination by a dentist is not feasible. Subsequently, each of these experts proposed a series of questions and items for each domain outlined in both forms. This led to the formation of another expert panel tasked with evaluating these questions, resulting in the initial draft of the Comprehensive Geriatric Oral Health Assessment Tool (CGOHAT). The questions underwent multiple rounds of revision guided by expert input. Additional details regarding the self‐assessment questions within the CGOHAT can be found in Table 1. Moreover, the domains and items featured in the clinical examination form are summarized in Table 2.

**Table 1:** Main domains and items of the self‐assessment oral health questionnaire.

Domains	Items
Edentulism and tooth loss	Number of remaining teeth
	Having removable dentures
	Partial
	Complete
	Satisfaction with denture
Dental caries	Pain or sensitivity
	While chewing
	While using sweets
	While drinking
	Having carious tooth
Periodontal health status	Bleeding
	Suppuration
	Inflammation
	Recession
	Tooth mobility
Oral lesions	Change in color
	Exophytic lesions
	Ulcers
Occlusal Problems	Having problem
Xerostomi	Dry mouth
	Burning mouth
Temporomandibular joint	Having pain
	Hearing sounds
Oral or prosthetic hygiene	Frequency
	Habits
	Removing dentures at night
Overall satisfaction	Very poor
	Poor
	Moderate
	Good
	Excellent

**Table 2:** Main domains and items of the clinical examination form.

Domains	Items
Dental examination	Number of natural teeth
	Having removable prosthetics
	Tooth examination
	*S*ound, no filling, fracture, or caries
	*D*ecayed
	*D*ecayed *F*illed (or single crowns), with decay
	*F*illed (or single crowns) without any decay
	*C*rown (bridge abutment, special crown, or veneer)
	*M*issing, as a result of caries
	*R*emained root
	*P*ontic (*M*issing replace with fixed prosthesis)
	*I*mplant
	*M*issing replace with removable *P*rosthesis
	rauma (fracture)
	*N*ot recorded
Periodontal examination	Periodontal pocket
	1–3.5 mm
	3.5–5.5 mm
	>5.5
	No pocket due to tooth loss
	Bleeding on probing
	Positive
	Negative
	No bleeding due to tooth loss
	Gingival recession
	Present calculus
Removable denture status	Hygiene
	Retention
	Attrition
Soft tissue examination	Lobulated tongue
	Mirror test
	Red and white lesions
	Exophytic lesions
	Ulcers
TMJ examination	Signs
	No signs
	Tenderness
	Symptoms
	Deviation
	Deflection
	Maximum mouth opening
	Clicking
	Crepitus

Abbreviation: TMJ, temporomandibular joint.

The combined set of questions included 38 questions in the clinical form and 50 questions in the self‐assessment form. An expert panel consisting of eight experienced dentists and physicians in the fields mentioned earlier was convened to assess the content and face validity of the CGOHAT. A qualitative assessment of face validity was conducted, leading to the revision of any questions with low face validity.

The content validity was evaluated using the content validity ratio (CVR) and the content validity index (CVI). For each question, these indices were calculated by having the experts complete a Likert scale (Zamanzadeh et al., 2015). Questions with CVI or CVR values below 0.8 were removed from the tool. Meanwhile, face validity was assessed through the input of an expert panel as well as geriatric subjects.

The CGOHAT was primarily designed and written in Persian, and the validity and reliability were evaluated using the Persian version. However, the English translation of CGOHAT is available in Supporting Information: Materials 1 and 2.

### 2.3 Pilot study and reliability

The pilot study was devised to assess both the face validity and the reliability of CGOHAT. The study participants were residents of the Kahrizak Charity Foundation (KCF) (Shoaee, Rezaie, et al., 2022), a nursing home located in Tehran, Iran, which houses approximately 900 elderly individuals and 600 disabled individuals (Shoaee, Rezaie, et al., 2022). A total of 84 subjects were selected through nonprobability judgmental sampling from two specific wards within KCF. The inclusion criteria for this study were as follows: (1) willingness to participate, (2) proficiency in the Persian language, (3) no oral examination‐related issues, and (4) absence of cognitive disorders.

The self‐assessment form was completed by the elderly individuals themselves, and a comprehensive protocol was developed for the oral health examination, guided by the expertise of an oral examination specialist and in accordance with the WHO oral health survey guidelines. Subsequently, the clinical forms were completed following a thorough oral examination conducted by two experienced dentists. Additionally, an interviewer administered the Geriatric Oral Health Assessment Index (GOHAI) questionnaire to each participant.

The internal consistency reliability was evaluated by calculating Cronbach's *α* coefficient for each domain (Devitt et al., 1998). Cronbach's *α* coefficient for each domain was: dental caries (*α* = .74), periodontal diseases (*α* = .78), edentulism and tooth loss (*α* = .74), oral lesions (*α* = .68), biting and chewing status (*α* = .76), xerostomia (*α* = .72), TMJ (*α* = .58), and oral or prosthetic hygiene (*α* = .67).

We evaluated the sensitivity, specificity, negative predictive value (NPV), positive predictive value (PPV), negative likelihood ratio (NLR), and positive likelihood ratio (PLR) of the self‐assessment questions in comparison with the clinical examination questions.

### 2.4 Ethical consideration

This study was approved by the Ethical Committee, Tehran University of Medical Sciences, Tehran, Iran, with code EC‐00291. Before data gathering, the participants were informed regarding the oral examination or self‐assessment questions. An informed consent was obtained from the participants and they were free to leave upon their intention.

## 3 RESULTS

A total of 84 residents from KCF participated in the pilot study, with an equal distribution of 42 males and 42 females. The participants had a mean age of 69.81 years (±4.12). Among them, 12 individuals (14.28%) had more than 12 years of education, while 72 individuals (85.72%) had less than 12 years of education. The average decayed, missing, filled teeth score was 29.92 (±4.63) and 24.21 (±9.65) for females and males, respectively. In terms of the GOHAI score, females had a mean score of 49.35 (±8.15), whereas males' mean score was 52.44 (±11.1). Notably, the GOHAI score exhibited a significant correlation solely with overall satisfaction with oral health status (*p* < .001); no other significant correlations were identified. Additional information about the participants' demographic characteristics can be found in Table 3.

**Table 3:** Demographic characteristics of the participants.

	Female (*n* = 42)	Male (*n* = 42)
Age (mean ± SD)	77.33 ± 8.70	64.45 ± 11.76
Education, years		
≤12	38 (90.48%)	34 (80.95%)
>12	4 (9.52%)	8 (19.05%)
Dental status		
Dentate	16 (38.1%)	22 (52.38%)
Edentulous	26 (61.9%)	20 (47.62%)
Number of remaining teeth (mean ± SD) [dentate participants]	5.75 ± 7.2	15.25 ± 10.2
DMFT (mean ± SD) [all participants]	29.92 ± 4.63	24.21 ± 9.65
DMFT (mean ± SD) [dentate participants]	26.68 ± 6.36	18.2 ± 9.52

Abbreviation: DMFT, decayed, missing, filled teeth.

The questions concerning periodontitis, gingivitis, red or white lesions, ulcers, filled teeth, and exophytic lesions demonstrated the highest specificity levels, with values of 100%, 100%, 91%, 87%, 81%, and 80%, respectively. However, the questions about the number of remaining teeth and the presence of decayed or filled teeth exhibited higher sensitivity, with values of 97%, 88%, and 75%, respectively. Only the questions related to having decayed teeth and the number of decayed teeth yielded an NPV below 70%. On the other hand, questions concerning the number of filled teeth, the presence of oral ulcers, and experiencing xerostomia yielded a PPV below 70%. A comparison of sensitivity, specificity, NPV, PPV, NLR, and PLR of the self‐assessment questions with the clinical questions is detailed in Table 4.

**Table 4:** Sensitivity, specificity, NPV, PPV, NLR, and PLR of self‐assessment questions in comparison with clinical examination.

Question	Specificity	Sensitivity	PLR	NLR	NPV	PPV	*N*
Number of remaining teeth	75%	97%	3.88	0.04	75%	97%	79
Number of decayed teeth	50%	88%	1.76	0.24	62%	81%	35
Number of filled teeth	81%	75%	3.94	0.30	90%	60%	30
Having decayed teeth	70%	33%	1.1	0.95	30%	72%	34
Having oral ulcer	87%	40%	3.07	0.68	94%	20%	68
Having xerostomia	41%	50%	0.84	1.21	70%	22%	69
Having periodontitis	100%	25%	–	0.75	72%	100%	82
Having gingivitis	100%	16%	–	0.84	54%	100%	84
Total	60.4% (95% CI: 57.53–93.46)	53% (95% CI: 27.76–78.23)	–	–	68.37% (95% CI: 51.34–85.40)	69% (95% CI: 41.54–96.45)	–

Abbreviations: CI, confidence interval; NLR, negative likelihood ratio; NPV, negative predictive value; PLR, positive likelihood ratio; PPV, positive predictive value.

The answer to the number of remaining teeth and the teeth count in clinical examination were further analyzed. The agreement and expected agreement of the answers were 93.15% and 73.29%, respectively, with a *κ* coefficient of 0.76 (*p* < *.*001). Further information is available in Table 5.

**Table 5:** Number of remaining teeth in clinical examination and self‐assessment questionnaire.

Clinical self‐assessment	0	1–9	10–19	20–32	Total
**0**	**48 (96%)**	0 (0%)	0 (0%)	1 (11.11%)	49 (62%)
**1–9**	1 (2%)	**11 (100%)**	4 (44%)	0 (0%)	16 (20%)
**10–19**	1 (2%)	0 (0%)	**4 (44%)**	1 (11%)	6 (7%)
**20–32**	0 (0%)	0 (0%)	1 (11%)	**7 (77%)**	8 (10%)
Total	50 (100%)	11 (100%)	9 (100%)	9 (100%)	79 (100%)

*p* Value (Fisher's exact test)	*p* Value (weighted *κ*)	Agreement	Expected agreement	*κ*	Standard error
<.001	<.001	93.15%	71.29%	0.7615	0.08

*Note*: Bold value indicate the clinical evaluation and the subject's answer reached the same results.

## 4 DISCUSSION

According to the new global oral health policy framework established by WHO, there are six key challenges to address (Benzian et al., 2021). These challenges encompass: “Inclusion and community engagement, prioritizing equity and social justice, addressing sugars as a major shared risk factor, implementing significant systemic reforms, improving data quality for informed decision‐making, and closing financing gaps” (Benzian et al., 2021). In line with the fifth component of this agenda, a concentrated effort is required to enhance data collection. These endeavors, combined with a comprehensive analysis of the collected data, will ultimately lead to evidence‐based decisions and policies, aligning with the core objective of this initiative.

Furthermore, the WHO's global oral health program emphatically emphasizes the importance of addressing the oral health needs of disadvantaged and vulnerable populations in both developed and developing countries (Petersen & Yamamoto, 2005). Establishing long‐term goals and overhauling current policies related to the oral health of the geriatric population necessitates an enhancement in data collection tools (Benzian et al., 2021; Petersen et al., 2010), which constitutes the primary objective of this study.

The development of CGOHAT yields a dual impact. First and foremost, it assists clinicians in conducting a more comprehensive evaluation of their elderly patients' oral health status, enabling the formulation of optimal treatment plans. While other oral health questionnaires and indices like the Oral Impact on Daily Performance, GOHAI, and OHAT may not serve as diagnostic tools, CGOHAT proves valuable to clinicians in the diagnostic process and subsequent treatment planning (Adulyanon, 1997; Amilani et al., 2020; Atchison & Dolan, 1990; Chalmers et al., 2005). Second, CGOHAT is a useful data‐gathering tool that helps policymakers to precisely recognize the main oral health issues of this vulnerable population.

Although the WHO oral health survey stands out as the most comprehensive OHAT, encompassing both self‐assessment and clinical evaluation, its scope, which covers age groups ranging from 5 to 74 years, results in the omission of certain specific oral health concerns pertinent to the geriatric population, such as xerostomia (Petersen & Baez, 2013). Xerostomia is linked to systemic conditions like diabetes mellitus and Sjogren syndrome, and it significantly influences oral health‐related quality of life among geriatric individuals (Hahnel et al., 2014; Mauri‐Obradors et al., 2017; Pérez‐González et al., 2021; Psianou et al., 2018).

Comparatively, CGOHAT provides a more comprehensive dentition status assessment and self‐assessment questions when juxtaposed with the self‐assessment questions of the WHO survey. Moreover, in the context of national surveys and scenarios where an oral examination by a dentist is unfeasible, the self‐assessment questions pertaining to remaining teeth count, decayed teeth count, periodontal health, and xerostomia can serve as valid substitutes for an oral examination. The domains of other geriatric oral health questionnaires are concisely outlined in Table 6, providing a basis for comparison with the domains of CGOHAT, as presented in Tables 1 and 2.

**Table 6:** A brief summary of the main domains of the available geriatric oral health questionnaires.

Brief Oral Health Status Examination
Lymph nodes examination Soft and hard tissue examination Saliva (effect on tissue) Condition of natural teeth Condition of artificial teeth Pairs of teeth in chewing position (natural or artificial) Oral hygiene
Dental impact of daily living
Appearance dimension Pain dimension Comfort dimension General performance Eating restriction
Geriatric Oral Health Assessment Index
Functional oral limitation Psychosocial limitation Appearance Oral pain Sensitivity
Oral Health Assessment Tool
Oral soft tissue assessment Natural teeth Dentures Oral cleanliness Dental pain
Oral Health Impact Profile
Functional limitation Physical pain Psychological discomfort Psychological disability Social disability Handicap

Our study was faced with certain limitations. As the participants of this study were residents of a nursing home, they cannot be regarded as the representative of the Iranian geriatric population. The most important limitation was the low number of dentate people. More than half of our study population were edentulous.

## 5 CONCLUSION

The well‐being of the geriatric population, both in terms of general health and oral health, is gaining increasing significance due to the ongoing demographic transition. This study has successfully created a reliable and valid tool for evaluating the oral health of this population. Future research and inquiries should be conducted to assess its practicality and potential in enhancing the diagnosis and formulation of treatment plans for this particular population.

## AUTHOR CONTRIBUTIONS

**Shervan Shoaee:** Conceptualization; tool development; data gathering; data analysis; interpreting the results; drafting the first manuscript; revising the manuscript. **Mohammad‐Hossein Heydari**: Tool development; data analysis; interpreting the results; drafting the first manuscript; revising the manuscript. **Hossein Hessari**: Tool development; data gathering; interpreting the results; revising the manuscript. **Neda Mehrdad**: Tool development; interpreting the results; revising the manuscript. **Laleh Khalilazar**: Tool development; interpreting the results; revising the manuscript. **Bahareh Hatami**: Tool development; interpreting the results; revising the manuscript. **Farshad Sharifi**: Tool development; interpreting the results; revising the manuscript.

## CONFLICT OF INTEREST STATEMENT

The authors declare no conflict of interest.

## Supporting information

Supporting information.

Supporting information.

## Data Availability

Data is available upon request from the corresponding author.
