# Supplementary material for: Development and initial validation of the Comprehensive Geriatric Oral Health Assessment Tool
Source: Clin Exp Dent Res. 2023 Sep 28;9(5):879–86. doi: 10.1002/cre2.791 (PMC10582220; doi:10.1002/cre2.791)
Supplement: Supplementary file 1 — Supporting information. [file CRE2-9-879-s002.docx]

|  |  | **Form No.** |  |  | **Examiner Code** |
| --- | --- | --- | --- | --- | --- |

| **Age:** | **Female□ Male□Gender:** | | **First Name:**  **Last Name:** |  |
| --- | --- | --- | --- | --- |
| **Dental Status** | | | |  |
| **Natural Teeth? 0. Yes 1. No** | | | |  |
| **Number of Natural Teeth**: □□ | | | |  |
| **Removable Denture? 0. No 1. Yes** | | | |  |
| **Crown** | | | |  |
| \| 28 \| 27 \| 26 \| 25 \| 24 \| 23 \| 22 \| 21 \|  \| 11 \| 12 \| 13 \| 14 \| 15 \| 16 \| 17 \| 18 \| \| --- \| --- \| --- \| --- \| --- \| --- \| --- \| --- \| --- \| --- \| --- \| --- \| --- \| --- \| --- \| --- \| --- \| \|  \|  \|  \|  \|  \|  \|  \|  \|  \|  \|  \|  \|  \|  \|  \|  \|  \| \|  \|  \|  \|  \|  \|  \|  \|  \|  \|  \|  \|  \|  \|  \|  \|  \|  \| \| 38 \| 37 \| 36 \| 35 \| 34 \| 33 \| 32 \| 31 \|  \| 41 \| 42 \| 43 \| 44 \| 45 \| 46 \| 47 \| 48 \| | | | |  |
| **S= S**ound, no filling, fracture or caries | | | | |
| **D= D**ecayed | | | | |
| **DF= D**ecayed **F**illed (or single crowns), with Decay | | | | |
| **F= F**illed (or single crowns) without any decay | | | | |
| **C= C**rown (Bridge abutment, special crown or veneer) | | | | |
| **M= M**issing, as a result of caries | | | | |
| **R= R**emained root | | | | |
| **P**= **P**ontic (Missing replace with fixed prosthesis) | | | | |
| **I= I**mplant | | | | |
| **M= M**issing replace with removable **P**rosthesis | | | | |
| **T= T**rauma (fracture) | | | | |
| **N= N**ot recorded | | | | |
| **Root** | | | |  |
| **No. of Posterior Decayed Root(s):**   \| **Upper Jaw** \|  \|  \| \| --- \| --- \| --- \| \|  \|  \| **Lower Jaw** \| | | \| **Upper Jaw** \|  \|  \| \| --- \| --- \| --- \| \|  \|  \| **Lower Jaw** \|   **No. of Posterior Decayed Root(s):** | |  |

| \| **Periodontal Status** \| \| --- \|   Periodontal Pocket:  0. Pocket depth of 1 to 3.5 mm (The first black indicator of periodontal probe is observed)  1. Pocket depth is 3.5 to 5.5 mm (Part of the first black indicator is observed)  2. Pocket depth is more than 5.5 mm (The first black indicator can’t be observed)  9. Examination can’t be performed. (The tooth is missing or it can’t be examined) | | | | | | | | | | | | | | |
| --- | --- | --- | --- | --- | --- | --- | --- | --- | --- | --- | --- | --- | --- | --- | --- |
| 27 | 26 | 25 | 24 | 23 | 22 | 21 |  | 11 | 12 | 13 | 14 | 15 | 16 | 17 |
|  |  |  |  |  |  |  |  |  |  |  |  |  |  |  |
|  |  |  |  |  |  |  |  |  |  |  |  |  |  |  |
| 37 | 36 | 35 | 34 | 33 | 32 | 31 |  | 41 | 42 | 43 | 44 | 45 | 46 | 47 |

| Bleeding on Probing   1. There is no bleeding on probing   1. There is bleeding on probing.  9. Examination can’t be performed. (The tooth is missing or it can’t be examined) | | | | | | | | | | | | | | |
| --- | --- | --- | --- | --- | --- | --- | --- | --- | --- | --- | --- | --- | --- | --- |
| 27 | 26 | 25 | 24 | 23 | 22 | 21 |  | 11 | 12 | 13 | 14 | 15 | 16 | 17 |
|  |  |  |  |  |  |  |  |  |  |  |  |  |  |  |
|  |  |  |  |  |  |  |  |  |  |  |  |  |  |  |
| 37 | 36 | 35 | 34 | 33 | 32 | 31 |  | 41 | 42 | 43 | 44 | 45 | 46 | 47 |

| **Removable Denture Hygiene Status** | | | | |
| --- | --- | --- | --- | --- |
|  | | | | |
| **Is there any food debris?**   1. **No** 2. **Yes** 3. **Not applicable** | **Is there any calculus?**   1. **No** 2. **Yes** 3. **Not applicable** | | **Is there any plaque?**   1. **No** 2. **Yes**   **9. Not applicable** | **Is there pigment?**   1. **No** 2. **Yes**   **9. Not Applicable** |
| **Oral mucosa** | | | | |
| \|  \| \| --- \| | | **Is the tongue lobulated?**   1. **No 1. Yes 9. Not applicable** | | |
| \|  \| \| --- \| | | **Does dental mirror stick to the buccal mucosa?**   1. **No 1. Yes 9. Not applicable** | | |
| \|  \| \| --- \| | | **White and red lesions?**   1. **No 1. Yes 9. Not applicable** | | |
| \|  \| \| --- \| | | **Is there any exophytic lesion in the mouth?**   1. **No 1. Yes (Because of denture) 2. No (For reasons other than denture)**   **9. Not applicable** | | |
| \|  \| \| --- \| | | **Is there any ulcer in the mouth?**   1. **No 1. Yes 9. Not applicable** | | |

| **Clinical Gingival Recession**   1. **No 1. Yes 9. Not applicable** | **Calculus**   1. **No 1. Yes 9. Not applicable** |
| --- | --- |

| **Removable Denture Status** | | |
| --- | --- | --- |
| **Denture Attrition**   1. **No** 2. **Low** 3. **High**   **9. Not applicable** | **Stability**   1. **Inappropriate** 2. **Appropriate**   **9. Not applicable** | **Retention**   1. **Inappropriate** 2. **Appropriate**   **9. Not applicable** |

| **Temporomandibular Joint (TMJ)** | |
| --- | --- |
| \|  \| \| --- \|   Signs:   1. No 2. Yes (Deviation, Clicking, Maximum mouth opening less than 30 mm (approximately the size of two fingers)) | Symptoms:   \|  \| \| --- \|  1. No 2. Yes (Tenderness) |
