# Supplementary material for: Development and initial validation of the Comprehensive Geriatric Oral Health Assessment Tool
Source: Clin Exp Dent Res. 2023 Sep 28;9(5):879–86. doi: 10.1002/cre2.791 (PMC10582220; doi:10.1002/cre2.791)
Supplement: Supplementary file 2 — Supporting information. [file CRE2-9-879-s001.docx]

| \|  \|  \| **Form No.** \|  \|  \| **Questioner Code** \| \| --- \| --- \| --- \| --- \| --- \| --- \| | |  |
| --- | --- | --- | --- | --- | --- | --- | --- | --- |
| **First Name:**  **Last Name:** | **Gender:**  **Female□ Male□** | **Age:** |
| **Partial Edentulism and Edentulism** | | |
| **1. How many natural teeth you have in your mouth?**   1. **I have all of my natural teeth.** 2. **I have no teeth.** 3. **I have 1 to 9 teeth.** 4. **I have 10 to 19 teeth.** 5. **I have 20 teeth or more.** 6. **I don’t know.** | | |
| 1. **Do you have removable denture?** 2. **Yes, and I use it.** 3. **Yes, but I don’t use it.** 4. **No**   **9. I don’t know.**  **If “Yes”, answer the questions No. 3 to 7.**  **If “No”, go to question No. 14.** | | |
| 1. **Do you have removable partial denture on your upper teeth?** 2. **Yes, and I use it.** 3. **Yes, but I don’t use it.** 4. **No.**   **9. I don’t know.** | | |
| 1. **Do you have removable partial denture on your lower teeth?** 2. **Yes, and I use it.** 3. **Yes, but I don’t use it.** 4. **No.**   **9. I don’t know.** | | |
| 1. **Do you have complete upper denture?**      1. **Yes, and I use it.** 2. **Yes, but I don’t use it.** 3. **No.**   **9. I don’t know.**   1. **Do you have complete lower denture?**      1. **Yes, and I use it.** 2. **Yes, but I don’t use it.** 3. **No.**   **9. I don’t know.** | | |
| **7. Are you satisfied with your denture?**  **1. Yes**  **2. No**  **9. I don’t know.**  **If “No”, answer the questions No. 8 to 13.**  **If “Yes” or “I don’t know”, go to the question No. 14.** | | |
| **8. I’m not satisfied with my denture because it’s loose.**   1. **Yes** 2. **No** | | |
| **9. I’m not satisfied with my denture, because it comes out of my mouth while eating or talking.**  **1. Yes**  **2. No** | | |
| **10. I’m not satisfied with my denture because I got mouth sores**.  **1. Yes**  **2. No** | | |
| 1. **I am not satisfied with my denture because I can’t properly bite with them.** 2. **Yes** 3. **No** | | |
| 1. **I’m not satisfied with my denture because I got dental attrition.** 2. **Yes** 3. **No** | | |
| 1. **I’m not satisfied with my denture because it’s not esthetically satisfying.** 2. **Yes** 3. **No** | | |
| 1. **Have you had removable denture in the past?** 2. **Yes** 3. **No** | | |
| **Dental Caries and Pulp Diseases** | | |
| ***If you have answered question No. 1 “I have no teeth”, please then DO NOT answer questions of this section.***  **15. Have you experienced tooth pain or tooth sensitivity in the past three months?**  **1. Yes**  **2. No**  **9. I don’t know.**  ***If “Yes”, answer the questions No. 16 to 19.***  ***If “No”, then go to the questions No. 21.*** | | |
| 1. **I have had tooth pain or sensitivity while chewing food.** 2. **Yes** 3. **No** 4. **I don’t know.** | | |
| 1. **I have had tooth pain or sensitivity when eating sweets.** 2. **Yes** 3. **No** 4. **I don’t know.** | | |
| 1. **I have had tooth pain or sensitivity when eating and drinking hot or cold foods.** 2. **Yes** 3. **No** 4. **I don’t know.** | | |
| 1. **I have had tooth pain or tooth sensitivity due to a carious tooth.** 2. **Yes** 3. **No** 4. **I don’t know.** | | |
| 1. **Tooth pain or sensitivity due to other reasons:** 2. **Yes** 3. **No** | | |
| **Gingival Disease** | | |
| ***If you have answered question No. 1 “I have no teeth”, please then DO NOT answer questions of this section.***   1. **Do you have a problem such as bleeding, pus discharge, swelling or recession in your gum?** 2. **Yes** 3. **No** 4. **I don’t know.**   **If “No”, go to the question No. 26.** | | |
| 1. **My gums bleed when brushing or flossing.** 2. **Yes** 3. **No** 4. **I don’t know.** | | |
| 1. **I have gum recession.** 2. **Yes** 3. **No** 4. **I don’t know.** | | |
| 1. **I have gum disease.** 2. **Yes** 3. **No** 4. **I don’t know.** | | |
| 1. **I feel the taste of blood or spontaneous bleeding in my mouth.** 2. **Yes** 3. **No** 4. **I don’t know.** | | |
| 1. **Do you have calculus on your teeth?** 2. **Yes** 3. **No** 4. **I don’t know.** | | |
| 1. **Do you have mobile teeth in your mouth?** 2. **Yes** 3. **No** 4. **I don’t know.** | | |
| **Oral lesions** | | |
| 1. **Do you have discoloration on your lips or inside your mouth?** 2. **Yes** 3. **No** 4. **I don’t know.** | | |
| 1. **Do you have bumps on your lips or inside your mouth?** 2. **Yes** 3. **No** 4. **I don’t know** | | |
| 1. **Do you have sores on your lips or inside your mouth?** 2. **Yes** 3. **No** 4. **I don’t know.** | | |
| **Dry and burning mouth** | | |
| 1. **Do you have a burning sensation in your mouth?** 2. **Yes, always.** 3. **Yes, sometimes.** 4. **No** 5. **I don’t know.** | | |
| 1. **Have you experienced dry mouth in the last month?** 2. **Yes, a lot.** 3. **Yes, a little.** 4. **No** 5. **I don’t know.** | | |
| **Occlusal Status** | | |
| 1. **Do you have trouble chewing food?** 2. **Yes** 3. **No** 4. **I don’t know.**   ***If “Yes”, answer the questions No. 34 to 38.***  ***If “No”, go to the question No. 37.*** | | |
| 1. **I have trouble chewing and swallowing all kinds of food:** 2. **Yes, always.** 3. **Yes, sometimes.** 4. **No** 5. **I don’t know.** | | |
| 1. **I have difficulty chewing and swallowing dry foods such as bread.** 2. **Yes** 3. **No** 4. **I don’t know.** | | |
| 1. **I have difficulty chewing and swallowing solid foods such as cooked meat.** 2. **Yes** 3. **No** 4. **I don’t know.** | | |
| 1. **I have difficulty chewing and swallowing soft and semi-solid foods such as mashed potatoes and cooked vegetables.** 2. **Yes** 3. **No** 4. **I don’t know.** | | |
| 1. **Can you bite an apple?** 2. **Yes** 3. **No** 4. **I don’t know.** | | |
| **Temporomandibular Joint (TMJ)** | | |
| 1. **Do you have pain in your jaw (in front of the ear) when you open and close your mouth?** 2. **Yes** 3. **No** 4. **I don’t know**. | | |
| **Hygiene** | | |
| 1. **(If you have natural teeth), do you use a toothbrush to clean your teeth?** 2. **Yes** 3. **No**   ***If “Yes”, answer the questions No. 41 and 42.***  ***If “No”, go to the question No. 43*** | | |
| 1. **If used, how often:** 2. **Once a month** 3. **A few times a month** 4. **Once a week** 5. **A few times a week** 6. **Once a day** 7. **A few times in a day** 8. **I don't know** | | |
| 1. **What other materials and equipment do you use to clean natural teeth?** 2. **dental floss** 3. **toothpick** 4. **mouthwash** 5. **inter dental brush** 6. **baking soda** 7. **coal powder** 8. **tooth brush** 9. **salt** 10. **Other: ____________** | | |
| 1. **if you have a removable denture, do you clean it?** 2. **Yes** 3. **No**   ***If “Yes”, answer the questions No. 44 and 45.***  ***If “No”, go to the question No. 46.*** | | |
| 1. **If you clean it, then how often?** 2. **Once a month** 3. **A few times a month** 4. **Once a week** 5. **A few times a week** 6. **Once a day** 7. **A few times in a day** 8. **I don't know.** | | |
| 1. **What materials and equipment do you use to clean your denture?** 2. **Only water** 3. **Water and Soap** 4. **Tooth brush** 5. **Tooth paste** 6. **Denture disinfection materials** 7. **Other: __________** | | |
| 1. **Do you remove your dentures at night?** 2. **Yes, always.** 3. **Yes, sometimes.** 4. **No** | | |
| **General assessment** | | |
| 1. **Are you satisfied with your oral health status?** 2. **Not at All** 3. **To some extent** 4. **Moderate** 5. **Satisfied** 6. **Completely Satisfied** | | |
